# Identifying Natural Bioactive Peptides from the Common Octopus (*Octopus vulgaris* Cuvier, 1797) Skin Mucus By-Products Using Proteogenomic Analysis

**DOI:** 10.3390/ijms24087145

**Published:** 2023-04-12

**Authors:** Sara Pérez-Polo, Md Abdus Shukur Imran, Sonia Dios, Jaime Pérez, Lorena Barros, Mónica Carrera, Camino Gestal

**Affiliations:** Instituto de Investigaciones Marinas (IIM), CSIC, Eduardo Cabello 6, 36208 Vigo, Spain

**Keywords:** *Octopus vulgaris*, skin mucus, proteomics, mass spectrometry, bioactive peptides, food

## Abstract

The common octopus is a cephalopod species subject to active fisheries, with great potential in the aquaculture and food industry, and which serves as a model species for biomedical and behavioral studies. The analysis of the skin mucus allows us to study their health in a non-invasive way, by using a hardly exploited discard of octopus in the fishing sector. A shotgun proteomics approach combined with liquid chromatography coupled with tandem mass spectrometry (LC-MS/MS) using an Orbitrap-Elite instrument was used to create a reference dataset from octopus skin mucus. The final proteome compilation was investigated by integrated in-silico studies, including Gene Ontology (GO), the Kyoto Encyclopedia of Genes and Genomes (KEGG) pathway, network studies, and prediction and characterization analysis of potential bioactive peptides. This work presents the first proteomic analysis of the common octopus skin mucus proteome. This library was created by merging 5937 identified spectra of 2038 different peptides. A total of 510 non-redundant proteins were identified. Obtained results show proteins closely related to the defense, which highlight the role of skin mucus as the first barrier of defense and the interaction with the environment. Finally, the potential of the bioactive peptides with antimicrobial properties, and their possible application in biomedicine, pharmaceutical, and nutraceutical industry was addressed.

## 1. Introduction

Cephalopoda is a class of exclusively marine mollusks, characterized by vertebrate-like eyes, a highly centralized nervous system, a wide variety of defense mechanisms, and unique social behavior [1]. 

One of the first lines of interaction with the environment in cephalopods, as it also is in fish, is the skin mucus, which may generate a first response to stressors agents [2,3]. This skin mucus is produced by goblet cells and mucocytes [4,5]. It is composed of water, electrolytes, epithelial and blood cells, mucins, and bioactive molecules produced by mucus-secreting cells [2,6]. Mucus plays a fundamental role in protecting against cold and freezing temperatures and is relevant in locomotion, recognition, osmoregulation, and respiration. Moreover, it is important to remark its defensive function, acting as the principal barrier against several stressors, including chemical, physical, and biological agents. [2,7,8].

Additionally, to this wide spectrum of functions, several studies provide evidence that skin mucus can act as a repository of numerous innate immune components, and the study of these components could be regarded as a low-invasive technique for the identification of health-related biomarkers [2,3,8,9]. Moreover, the skin mucus of different mollusks has been described as having antimicrobial and antioxidant properties, among others [2,10,11]. However, very little is known about the mucosal immune system of cephalopods, and the properties of its skin mucus deserve to be explored.

Octopuses and squids are cephalopods of great importance in the food industry, since they are considered a very complete food and are highly appreciated by a large number of countries and cultures. Among them stands out the common octopus, *Octopus vulgaris*, Cuvier, 1797, which is a benthic, neritic species with great biological and economic value.

Currently, this species is overexploited, especially in Galicia (NW Spain). Due to its fishing interest, protecting this resource while supplying the strong demand of the food market is a necessity. The development of efficient and sustainable aquaculture of the common octopus, which also guarantees animal welfare, is essential for this purpose.

Significant progress has been made in octopus farming [12] but this aquaculture practice is still in an experimental period, and efforts to improve it and produce it on a commercial scale are still ongoing.

Prevention as well as the fight against pathogens, adverse conditions, and external aggressions, as a strategy to improve their health and welfare [13], which in turn will have an impact on the quality and safety of octopus as a food product, is a key point where the skin mucus can play an important role by providing a low-invasive approach to study the octopus health status under aquaculture conditions.

Moreover, seasonality, geographical location, and even maturity status or stress situations of *O. vulgaris* (both in natural populations and aquaculture-confined specimens) could be involved in the differences related to the presence and quantity of the different proteins and bioactive compounds identified in the skin mucus. Therefore, the study of the composition of *O. vulgaris* skin mucus proteome can provide important information on the environmental conditions surrounding the species without damaging the specimens, including its physiological status and immune defense capability.

The present work deals for the first time with the global characterization of the *O. vulgaris* skin mucus proteome using a shotgun proteomic approach. The aim is to extract information about the defensive mechanisms that mucus components can provide as a first physical barrier, and to identify the potential bioactive peptides found in the mucus components. Apart from participating in the immune defense of the octopus, and therefore in the octopus health, these bioactive compounds could have infinite applications in the biomedicine, pharmaceutical, and nutraceutical industries, so their knowledge could be of great value in these sectors.

## 2. Results

### 2.1. Protein Concentration by BCA and SDS-PAGE

The protein concentrations of the samples were calculated using the bicinchoninic acid method (BCA) based on the standard curve obtained (Table 1). The concentrations in the samples were in a range from 0.07 µg/µL (O4) to 2.43 µg/µL (O1).

**Figure 1:**
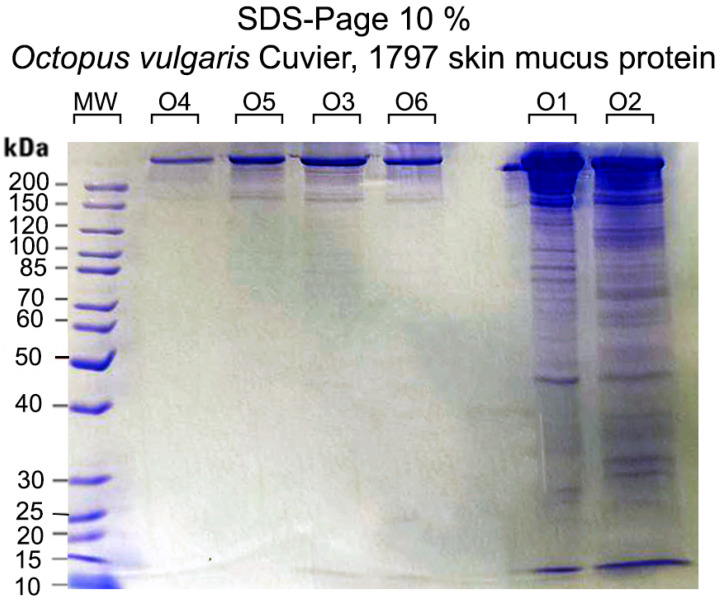
SDS-PAGE 10% profiles of the extracted proteins of *O. vulgaris* skin mucus samples (O1–O6). MW denotes molecular weight.

Proteins extracted from the mucus of six specimens (O1–O6) were separated based on their molecular weight using SDS-PAGE 10% to display the corresponding electrophoretic profile of each sample (Figure 1). The extracts of different samples show variability in the separation of the proteins, as it is observed in the polyacrylamide gel (Figure 1). The O1 and O2 profiles displayed a complex band pattern due to a higher protein concentration. Sample O4 was discarded for the rest of the analysis, due to the low protein concentration obtained. 

### 2.2. Mass Spectrometry (MS)

To the best of our knowledge, this is the first proteomics analysis for the common octopus skin mucus proteome. It was established by merging 5937 identified spectra (Peptide Spectrum Matches, PSMs) from 2038 different peptides (Table S1 in Supplementary Materials). Supplementary Table S2 summarizes a total of 510 non-redundant proteins identified from all the different samples (n = 5), based on the LC-MS/MS analysis and the search engine SEQUEST-HT algorithm of the tryptic digestions for the global protein extractions. Raw data and analysis outputs are publicly available in the MassIVE data repository (https://massive.ucsd.edu/, accessed on 15 July 2022) (Reference: MSV000089888) and the ProteomeXchange database (https://www.proteomexchange.org/, accessed on 15 July 2022) under accession number PXD035356.

Of the 510 proteins detected (Supplementary Table S2), a total of 313 were assigned to the species *O. vulgaris*. A total of 112 uncharacterized proteins were observed, 47 of them related to *O. vulgaris*. The final global dataset of the *O. vulgaris* skin mucus proteome was subsequently investigated by protein-based bioinformatics, including gene ontologies, pathways, network analyses, and by the prediction of potential bioactive peptides to gather more functional insights of *O. vulgaris* skin mucus.

### 2.3. Label-Free Quantification (LFQ) of O. vulgaris Mucus Samples

**Figure 2:**
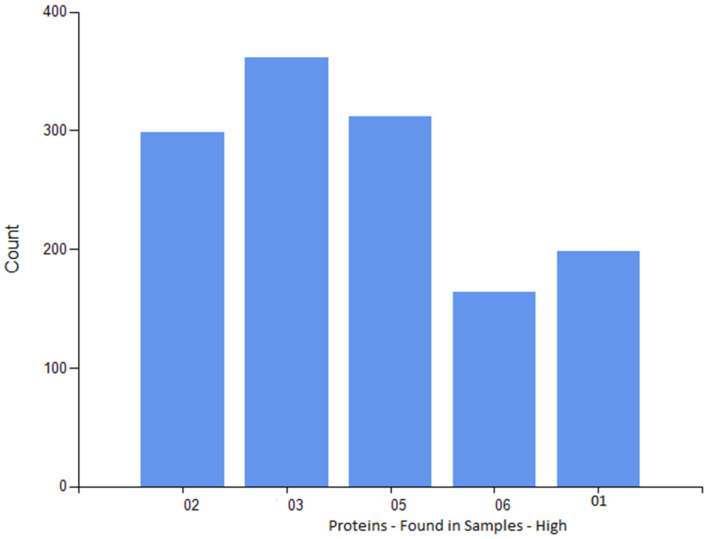
Distribution of the high-abundance proteins for *O. vulgaris* mucus samples (O1, O2, O3, O5, and O6) determined by LFQ.

Relative label-free quantification of each *O. vulgaris* mucus sample (O1, O2, O3, O5, and O6) was also performed to determine the protein abundance of each sample. Supplementary Table S1 contains these results. High-abundance proteins for each sample were analyzed and compared. Figure 2 shows the distribution of the high-abundance proteins detected for each *O. vulgaris* mucus sample. As was demonstrated in Figure 2, the majority of the high-abundance proteins were detected in the O3 sample.

**Figure 3:**
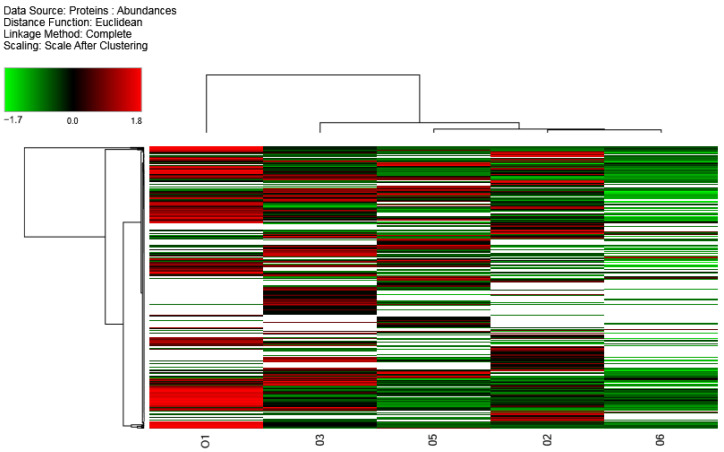
Heatmap from the shotgun proteomics analysis of the different mucus samples (O1, O2, O3, O5, and O6). Every bar corresponds to the presence or absence of a particular protein. Euclidean hierarchical distances were sorted for all samples.

The distribution of the high-abundance proteins for all samples analyzed by LFQ was also illustrated in a heatmap diagram in Figure 3. Euclidean hierarchical distance was used to differentiate the two main clusters.; Cluster A (sample O1) and Cluster B (samples O2, O3, O5, and O6).

### 2.4. Functional Analysis: Gene Ontologies and Pathways Analysis

**Figure 4:**
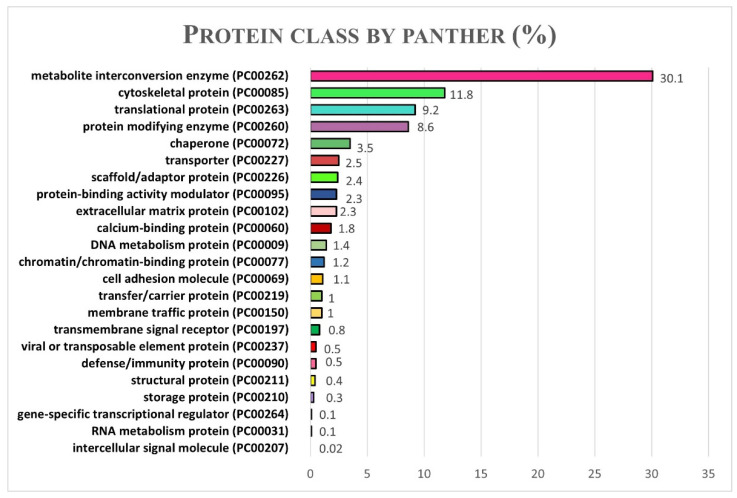
Protein Class of *O. vulgaris* skin mucus proteome identified by shotgun proteomics and categorized by PANTHER using the gene name as input for the software (http://pantherdb.org, accessed on 4 June 2022).

PANTHER analysis was realized using the gene name (considering all non-redundant proteins) revealing the presence of 11 different molecular functions (Figure S1), 16 different biological processes (Figure S2), and 23 different protein classes (Figure 4) in *O. vulgaris* skin mucus proteome.

For each of the PANTHER groups identified, the PANTHER subgroups classification is listed below and included in Supplementary Table S3.

In the Protein Class classification (Figure 4), the most prominent classes were metabolite interconversion enzyme (30.1%), cytoskeletal protein (11.8%), translational protein (9.2%), protein modifying enzyme (8.6%), and chaperones (3.5%).

Within the metabolite interconversion enzyme, different enzyme groups were observed including carbohydrate kinases, aldolases, isomerases, dehydrogenases, hydrolases, glycosidases, transferases, oxidases, glucosidases, peroxidases, mutases, dehydratases, phospholiases, isomerases, and deaminases. Within the cytoskeletal protein group, non-motor actin binding protein, intermediate filament binding protein, actin or actin-binding cytoskeletal protein, tubulins, and microtubule or microtubule-binding cytoskeletal protein were observed. In the translational protein category, elongation factor and ribosomal protein were found. Within the protein modifying enzyme category, proteases, metalloproteases, serine proteases, ubiquitin-protein ligases, non-receptor serine/threonine protein kinases, and cysteine proteases were represented. In the chaperone group, chaperones, chaperonins, and Hsp90 and Hsp70 family chaperones (heat shock proteins) were detected.

The defense/immunity protein category was observed in a small percentage (0.5%) in comparison with other categories and contained the subclass complement component.

A molecular function analysis (Figure S1) revealed a significant percentage of proteins with catalytic activity (36.3%), binding (34.9%), and structural molecule activity (8.4%). As for the Biological Processes classification (Figure S2), the most remarkable categories were cellular process (59.3%), metabolic process (38.1%), biological regulation (14.9%), localization (12.6%), response to stimulus (9.5%), developmental process (6.9%), and signaling (4.1%).

For the analysis of KEGG pathways, DAVID program version 6.8 (https://david.ncifcrf.gov/home.jsp, accessed on 6 June 2022) was used including the genome background of *O. bimaculoides*, as the phylogenetically closest cephalopod species to *O. vulgaris*.

The KEGG results (Table 2) showed that most of the identified proteins were involved in the phagosome (endocytosis) and ribosome (amino peptides synthesis) pathways.

The DAVID software was also used to study the functional domains by InterPro (Table 3). The results emphasize the functional domains that corresponded to the von Willebrand factor protein domains, Lamin tail domain, lipid transport proteins, glycoside hydrolases, metallopeptidases, and EF-hand domains. An immunoglobulins-like domain was also observed, but with less *p*-value.

### 2.5. Network Analysis

**Figure 5:**
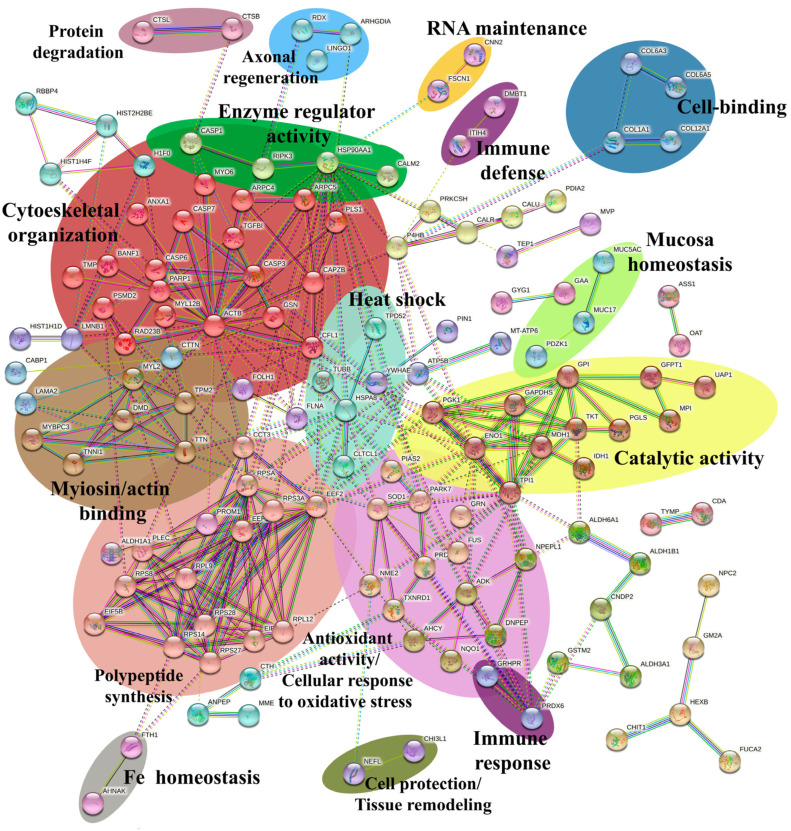
Protein network for the *O. vulgaris* skin mucus proteome with STRING (https://string-db.org, accessed on 8 June 2022) software. Nodes represent the proteins and the interactions between proteins are represented with continuous lines if referred to direct interactions (physical) or with dotted lines if referred to indirect interactions (functional).

A network analysis was performed using the STRING software (v.11.5) (https://string-db.org/, accessed on 8 June 2022) by analyzing all the proteins identified in this study and comparing them with the genome of *Octopus* spp. and *Homo sapiens* organisms since *O. vulgaris* is not available in the STRING software. *Octopus* spp. was considered as the genetically closest group. *H. sapiens* was taken into account as the group with the most information regarding genes and homologous in common with our results. Figure 5 shows the results obtained with an MCL = 2 inflation clustering, where 204 nodes (proteins) and 322 interactions (edges) are displayed. From the cluster analysis, 33 significant clusters of interactions between nodes were obtained, where only the most relevant (n = 16) were highlighted according to the abundance of nodes involved or their biological relevance (Figure 5).

To the best of our knowledge, this network represents the first interactomic map for the *O. vulgaris* skin mucus proteome. The most relevant sub-networks in terms of their number of nodes are involved in the structural constituent of muscle and cytoskeletal organization (in red, 19 nodes), the synthesis of polypeptides (in salmon, 14 nodes), in catalytic activity (in yellow, 12 nodes), and the cellular response to oxidation stress (in pink, 8 nodes).

Other sub-networks with fewer nodes but of great biological importance are related to myosin/actin binding (in brown, 8 nodes), immune defense/response (in violet, 2 nodes each), enzyme regulator activity (in green, 4 nodes), and heat shock (in aquamarine, 4 nodes). Additionally, various sub-networks of 2-3 nodes are involved in axonal regeneration (in blue), mucosa homeostasis (in lime), cell binding (in dark blue), cell protection/tissue remodeling (in olive), RNA maintenance (in orange), Fe homeostasis (in grey), and protein degradation (in purple).

### 2.6. Potential Bioactive Peptides Identification

Enzyme action is necessary for the activation of bioactive peptides. For this reason, the prediction of bioactive peptides was conducted by in-silico enzymatic digestions (trypsin and pepsin) using MS-Digest software, with a minimum of six residues per peptide and no missed cleavages (Supplementary Table S4). Pepsin cleaves proteins at residues Phe, Tyr, Trp, and Leu and trypsin cleaves the proteins at Lys and Arg residues [14].

A total of 15,356 different peptides were released with pepsin digestion (6–44 amino acid residues) and were classified by the software PeptideRanker (http://distilldeep.ucd.ie/PeptideRanker/, accessed on 10 June 2022) using the N-to-1 neural network probability [15] to predict peptides that may be more bioactive. The results obtained for all peptides are shown in Supplementary Table S4 and peptides with a score greater than 0.9 were chosen as potential bioactive peptides (Supplementary Table S5), selecting a total of 27 peptides (7–32 amino acid residues). Such peptides corresponded to metalloendopeptidases, progranulin, neuroblast differentiation-associated protein AHNAK, MAM and LDL-receptor, heat shock 71 kDa, collagen, hemocyanin subunit, titin, mucin, and different uncharacterized proteins. Compared with the BIOPEP-UWM database, no bioactive peptides were identified, probably because no information on *O. vulgaris* is available in this database.

The enzymatic digestion using trypsin released a total of 23,995 different peptides (6–44 amino acid residues). Using a PeptideRanker score higher than 0.9, a total of 230 tryptic peptides (6–36 amino acid residues) were selected as potential bioactive peptides (Supplementary Table S5). The majority of such peptides corresponded to collagen, metalloendopeptidases, choline transporters, prominins, mucins, hemocyanins, MAM and LDL-receptor class A proteins, hyaluronidases, tetraspanin, SCO-spondin proteins, myosin, PDZ and LIM domain, RING type proteins, lysozymes, ribosomal proteins, factor H proteins, H^+^-transporting two-sector ATPase proteins, and different uncharacterized proteins. Neural cell-related peptides and immunoglobulins-like peptides were observed in lower proportions.

Antimicrobial peptides (AMPs) were identified using the CAMP (Collection of Anti-Microbial Peptides) database (http://www.bicnirrh.res.in/antimicrobial/, accessed on 19 June 2022) and applying the DAC score (Discriminate Analysis Classifier score). Supplementary Table S5 summarizes these results, with a total of 18 pepsin peptides and 81 tryptic peptides with antimicrobial properties. Most of them are encrypted in the mucin parent protein (seven trypsin and one pepsin AMPs), in the hemocyanin parent protein (four trypsin and one pepsin AMPs), in the MAM-LDL receptor parent protein (two trypsin and one pepsin AMPs), in the SCO-spin parent protein (three tryptic AMPs), in the metalloendopeptidase parent protein (two pepsin AMPs), in the prominin parent protein (two tryptic and pepsin AMPs), in PDZ and LIM domain parent protein (two tryptic AMPs), in the myosin parent protein (one tryptic AMP), in the collagen parent protein (one tryptic AMP), in choline transporter parent protein (one tryptic AMP), in tetraspanin parent protein (one tryptic AMP), in Ring type parent protein (one tryptic AMP), in ribosomal parent protein (one tryptic AMP), in H^+^-transporting two-sector ATPase parent protein (one tryptic AMP), in lysozyme parent protein (one tryptic AMP), in titin parent protein (one pepsin AMP), in progranulin parent protein (one pepsin AMP), and in heat shock 71 kDa parent protein (one pepsin AMP).

Many potential bioactive peptides were also observed in uncharacterized proteins and many of them with AMPs.

## 3. Discussion

As far as we know, this is the first time that a global dataset of the proteins and peptides contained in the common octopus skin mucus has been obtained. These results provide an overview of its composition and how the proteins interact with each other and with the environment.

Although recent advances in next-generation sequencing have enabled the knowledge of the genome of *O. bimaculoides* [16], *Callistoctopus minor* [17], and *Euprymna scolopes* [18], the scarcity of public protein and gene databases is one of the major limitations in working with non-model organisms. In fact, the genome of *O. vulgaris,* although it is currently in progress, has not been completely sequenced and accurately annotated [19]. This is the reason why protein identification was conducted by using two databases: the Cephalopoda protein dataset from UniProtKB database, and the UniGene transcriptome database of *O. vulgaris* paralarvae [20]. However, despite using both databases, the results obtained showed 112 uncharacterized proteins while only 313 were recognized as proteins for the *O. vulgaris* species.

The common octopus is a species with great food importance, since it is a complete food with great nutritional value and it is highly appreciated. Due to the high scientific interest and high commercial value of the common octopus, several transcriptomic and proteomic studies have been published, including adult specific organs [21,22,23] and paralarvae under different growth conditions [24,25,26]. However, the biological processes of this species are still not well understood due in part to this lack of genome sequencing and accurate annotation. In fact, this is the first time that the proteins of the skin mucus have been specifically analyzed, and thus it is reasonable that a complete match with the currently published databases was not obtained. Fortunately, many of the peptides are conserved in different similar species, and hence a good contrast of the results has been possible.

Within the proteins and peptides identified, a large proportion of mucins and collagen were observed. Since mucus is mainly composed of mucilaginous elements [2,6,27], which help to maintain homeostasis and to prevent desiccation, this result was somehow an expected outcome.

Additionally, a high proportion of hemocyanin fragments and peptides was found. The presence of hemocyanin in the mucus could be related to a potential role of gas interchange carried out by the skin, even when respiration is mainly accomplished by the gills [28].

The presence of muscular tissue proteins such as myosin, paramyosin, and tropomyosin was also observed. This is probably related to an unintentional detachment of muscle tissue from the skin.

The results obtained by the PANTHER classification indicated that the principal molecular functions of the skin mucus common octopus proteins were catalytic activity, binding, and structural molecule activity. Proteins with catalytic activity function as enzymes-catalysts that increase the rate of virtually all the chemical reactions within cells. On the other hand, proteins with a binding function are referred to any protein that acts as an agent to bind two or more molecules together. Therefore, the presence of these binding proteins could explain the cohesion and the maintenance of the mucus structure, which is also important for good homeostasis and the role of the mucus as a protective barrier. Regarding biological processes, mucus proteins were mainly involved in the cellular process, metabolic process, biological regulation, localization, response to stimulus, developmental process, and signaling. These results could reflect the relationship of the mucus with the environment, as these proteins focus on processes of the response to external factors, signaling, and recognition, or on processes of maintenance and regulation of the mucus itself, also in accordance with the Molecular Functions categories obtained. Protein classes were represented by the metabolite interconversion enzyme, cytoskeletal protein, translational protein, protein modifying enzyme, structural protein, and chaperones. These proteins are classes of proteins related to the cellular structure of the mucus or related to enzyme activity, supporting the importance of the enzyme activity observed in the Molecular Function and Biological Processes classification. Finally, the presence of chaperones seems to indicate that mucus plays a key role in the immune response, which is discussed in more detail below.

Mucus is of great biological importance in the defense against both physical and biological factors [2,7,8]. As the first line of defense for the common octopus, mucus can confront physical variations such as a sudden increase or decrease in ambient temperature. Our results showed that some proteins within the Protein Class can act against this thermal stress, as in the case of heat shock proteins. Heat shock proteins are molecular chaperones implicated in a wide variety of cellular processes, including protection of the proteome from stress, the folding and transport of newly synthesized polypeptides, activation of proteolysis of misfolded proteins, and the formation and dissociation of protein complexes [29,30]. This chaperone activity was also identified in the network analysis in those processes related to heat shock and response to thermal stress. Finally, it is a protein with a high potential to contain bioactive peptides with antimicrobial properties. Consequently, heat shock proteins seem to play an important role in the response to temperature changes for the common octopus and its presence in the mucus confirms the importance of the mucus as a barrier against physical variations in the environment.

Following the PANTHER results obtained, a relevant presence of histones has been observed. Histones are DNA-binding proteins mainly participating in the nucleosomes wrapped inside the nucleus [31]. However, in other invertebrates or fish studies, histones were present in the cytoplasm and extracellular fluids and showed antimicrobial activity against bacteria, viruses, parasites, and fungi [32,33]. Almeida et al. [34] found large amounts of histones and histone-related AMPs in the saliva of *O. vulgaris*, which reinforced the hypothesis as proteins with a defensive function. For the histones studied in this work, most of the sequences obtained in the MS-Digest showed antimicrobial activity.

Another protein related to the innate immune response is the complement factor H-like, which was found within the stimulus response and the immune system process groups in the PANTHER analysis (within the complement components). Factor H is a soluble glycoprotein whose principal function is to regulate the alternative pathway of the complement system, ensuring that the complement system is directed towards pathogens or other dangerous material and exerts its protective action on self-cells [35] and helps in the correct cellular recognition of pathogens in the organism. Furthermore, it has a high potential to contain bioactive peptides (scores 0.938016 and 0.93163 after trypsin digestion).

The protein leucine-rich repeat, involved in immune response mechanisms, has also been identified. This protein had a high score (<0.90) in the Peptide Ranker results, which indicates a high probability of containing bioactive peptides. Leucine-rich repeats appear to provide a structural framework for the formation of protein–protein interactions [36]. Proteins containing leucine-rich repeats include tyrosine kinase receptors, cell-adhesion molecules, virulence factors, and extracellular matrix-binding glycoproteins, and besides immune response, are involved in a variety of biological processes, including signal transduction, cell adhesion, DNA repair, recombination, transcription, RNA processing, disease resistance, and apoptosis [37].

Previous studies highlighted the importance of mucus in the immune response, which has been determined as acting as a repository of numerous innate immune components such as molecules with antimicrobial activity [2,3,8,9]. The results obtained in this work support as well the presence of innate immune components and the fundamental role that mucus could play as a defensive system.

The presence of bioactive peptides as key proteins in the skin mucus, playing an important role in the defense against microorganisms and pathogens, has been identified and described in several studies, mainly focused on fish [38,39]. Additionally, different studies pointed out the properties of marine bioactive peptides as further medical, pharmaceutical, nutraceutical, and cosmetic applications. Putative antihypertensive, drug-delivering, and antiparasitic bioactive peptides were highly abundant within the mucus peptides on mollusks [40]. Particularly, antimicrobial peptides are a group of molecules produced by many tissues and cell types in a variety of species in contact with infectious microorganisms. The high potential of antimicrobial bioactive peptides in abundant compounds such as mucin and hemocyanin identified in the skin mucus proteome seems to suggest a considerable antimicrobial efficacy of mucus. In this sense, the common octopus could probably exploit these secreted antimicrobial bioactive peptides as part of an innate defense mechanism [34,41,42].

The in-silico analysis for the identification of bioactive peptides showed however many uncharacterized proteins containing bioactive peptides with a putative antimicrobial capacity. Further studies on the characterization of these proteins in the common octopus would be of great interest to increase the knowledge on potential bioactive peptides and its role on the defense against pathogens.

Besides antimicrobial capacity, the bioactive peptides found in the octopus skin mucus could also play other applications and functions of great interest. Potential pepsin and tryptic bioactive hemocyanin peptides were found in this study. Hemocyanin proteins are known to have important antimicrobial, binding, antiviral, and antitumor functions [43]. Other studies have observed the presence of active biopeptides associated with hemocyanin, highlighting their potential application as an antitumor therapy for cancer cells [44]. Because of its immunogenicity and peculiar quaternary structure, hemocyanin has been used as a scaffold for the conjugation of small molecules or short peptides, when raising anti-epitope antibodies or a possible model for the semi-synthesis of blood substitutes [45]. The hemocyanin peptides obtained in this study could be artificially synthesized and functionally evaluated as an antitumor therapy.

A large number of potential pepsin and tryptic bioactive Mucin-19 peptides was observed. Mucins have the ability of not only showing antibacterial activity but also acting as a protective surfactant to cover biomaterials to suppress the immunological response and have an important role as the first step of nutrient and drug diffusion [46]. Thus, the study of these peptides could be of interest, especially in drug delivery applications.

Potential pepsin bioactive Titin peptide was also observed. This peptide may be a potential biomarker of changes in quality and health in *O. vulgaris* as observed in other cephalopods [44].

The potential tryptic bioactive SCO-spondin-like peptide can be used as a bio-adhesive, since it forms strong attachments and resists degradation [47]. Other peptides such as potential tryptic and pepsin bioactive prominin peptide, potential pepsin bioactive metalloendopeptidase peptides, potential pepsin and tryptic bioactive MAM and LDL-receptor class A domain-containing protein peptides, or potential pepsin and tryptic bioactive progranulin peptides also have other potential functions in addition to their antimicrobial activity, although there is little information on their potential applications, which postulates them as possible candidates for future research.

A future line of research could focus on the importance of identifying these compounds and developing future experiments to confirm their functionality and determine their possible applicability in various fields, both as a sustainable use of fishing discards and as biomarkers of health in natural or cultured octopus populations, as well as in biomedicine, pharmacology, or cosmetics.

## 4. Materials and Methods

### 4.1. Animal Capture and Maintenance

Six specimens of *O. vulgaris* with an average weight of 1 kg (980–1300 g) were collected using fishing cages by professional certificated fishermen at the Ría de Vigo, Spain (24°14.09′ N, 8°47.18′ W) and were transported in proper containers to the Experimental Culture Facilities of IIM-CSIC, institution which is registered as “User and breeding center on animal experimentation” ES360570202001. Transport, housing, and handling were carried out following the principles of animal welfare. Following the European Directive 2010/63/EU, special attention has been paid to the 3Rs strategy (Reduce, Refine, Reuse), reducing the number of animals used in the experimental assay until the essential for having statistical significance. The experiments were also approved by the Ethic Committee of the Competent Authority (CEIBA2014-0108; ES360570202001/17/EDUCFORM 07/CGM02).

Specimens were maintained individually in tanks of 500 L of filtered aerated seawater at 15 ± 1 °C with a continuous re-circulating flow. The photoperiod was of 12 h light:12 h dark and cleaning and water parameters checking were performed daily. Food, consisting of frozen fish and mussels, was also supplied every day.

### 4.2. Sample Collection

Sampling procedures were always performed under anesthetic conditions using a mix of C_2_H_5_OH (1%) and MgCl_2_ (0.011 g mL^−1^) dissolved in seawater to reduce distress [46]. Skin mucus samples were collected from octopus specimens using the method of Guardiola et al. [8] with some modifications. Briefly, the skin mucus was collected from each animal by gentle squeezing and scraping the skin surface of each arm using a cell scraper and manually. The samples were vigorously shaken and stored at −80 °C until use.

**Figure 6:**
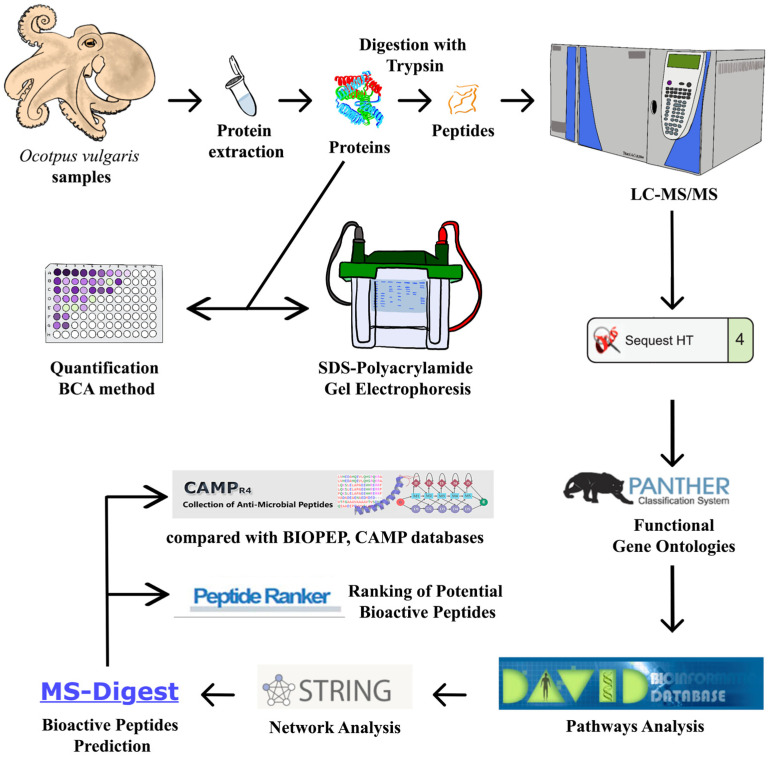
Workflow used to perform the extraction, quantification, and digestion of proteins. On the other hand, the steps followed the LC-MS/MS analysis for the bioinformatics study of the obtained proteins are shown.

A chart of methodology workflow from *O. vulgaris* skin mucus sampling to the final bioactive peptides identification is shown in Figure 6.

### 4.3. Skin Mucus Protein Extraction

A total of 1 mL of *O. vulgaris* skin mucus per sample was homogenized in 3 mL of lysis buffer (10 mM Tris-HCl pH 7.2 with 5 mM of phenylmethylsulfonyl fluoride (PMSF)) on ice using a vortex. After that, the samples were centrifuged at 40,000× *g* for 20 min at 4 °C in a centrifuge (Avanti JXN-26, Beckman Coulter, Palo Alto, CA, USA). The supernatant proteins were recovered and stored at −80 °C until used. Protein concentration was determined by the bicinchoninic acid (BCA) method (Thermo Fisher Scientific, San Jose, CA, USA).

### 4.4. SDS-Polyacrylamide Gel Electrophoresis (SDS-PAGE)

Skin mucus proteins were separated on 10% (*v*/*v*) polyacrylamide gels (acrylamide/N, N0-ethylene-bis-acrylamide, 200:1). A total of 10.3–20 µg for the sample of proteins in Laemmli buffer was boiled for 5 min at 100 °C and centrifuged at 10,000× *g* for 2 min at 4 °C. The samples were then separated per well in a Mini-PROTEAN 3 cell (Bio-Rad, Hercules, CA, USA). A PageRuler unstained protein ladder was also used as the molecular weight (MW) indicator (Thermo Fisher Scientific, San Jose, CA, USA). Running conditions were 80 V for the first 20 min and then 150 V until the end of the electrophoresis. Gels were stained overnight with Coomassie Brilliant Blue R-250 (VWR International, Radnor, PA, USA), unstained by using a solution composed of 25% ethanol and 8% acetic acid, washed with 50% methanol (*v*/*v*), and scanned at 200 dpi.

### 4.5. Protein Digestion with Trypsin

Protein digestion was performed with trypsin as described by Carrera et al. [48]. Briefly, a total of 100 µg of *O. vulgaris* skin mucus protein extract of each sample was lyophilized using a SpeedVac concentrator (Gyrozen). The samples were then denatured in 8 M urea with 25 mM of ammonium bicarbonate pH 8.0 and reduced with 9 mM DTT (DL-dithiothreitol) 98% (HPLC) (Roche, Mannheim, Germany, EU) for 45 min at 56 °C. The samples were alkylated with 50 mM iodoacetamide (Thermo Fisher Scientific) in MilliQ water for 60 min at room temperature in the dark. Samples were next diluted 4-fold with 25 mM ammonium bicarbonate pH 8.0 to decrease the urea concentration. Proteins were finally digested with trypsin (Promega Madison, WI, USA) (1:100 protease-to-protein ratio) overnight at 37 °C. The reaction was stopped with 5% formic acid (FA) and samples were purified for MS analysis using the C18 MicroSpin™ column (The Nest Group, Southborough, MA, USA).

### 4.6. LC-MS/MS

Each sample was reconstituted in 0.1% FA, sonicated, and 1–2 µg was transferred to MS vials and analyzed by liquid chromatography–tandem mass spectrometry (LC-MS/MS) using a Proxeon EASY-nLC II liquid chromatography system (Thermo Fisher Scientific, San Jose, CA, USA) coupled with an LTQ-Orbitrap Elite mass spectrometer (Thermo Fisher Scientific). Peptide separation (1 µg) was performed on a reverse phase (RP) column (EASY-Spray column, 50 cm 75 µm ID, PepMap C18, 2 µm particles, 100 Å pore size, Thermo Fisher Scientific) with a 10 mm pre-column (Accucore XL C18, Thermo Fisher Scientific) using 0.1% FA (mobile phase A) and 98% acetonitrile (98% ACN) with 0.1% FA (mobile phase B). A 120 min linear gradient from 5 to 35% B, at a flow rate of 300 nL min^−1^ was used. A spray voltage of 1.95 kV and a capillary temperature of 230 °C were used for ionization. The peptides were analyzed in positive mode (1 µscan; 400–1600 amu), followed by 10 data-dependent higher energy collision-induced dissociation (HCD) MS/MS scans (1 µscans), using a normalized collision energy of 35% and an isolation width of 3 amu. Dynamic exclusion for 30 s after the second fragmentation event was applied and unassigned charged ions were excluded from the analysis. A total of five samples (n = 5) were independently analyzed.

### 4.7. Processing of the Mass Spectrometry Data

The mass spectra obtained in the LTQ-Orbitrap Elite instrument was compared and validated with a theoretical mass spectra by using the Cephalopoda protein dataset from the UniProtKB database (including canonical and isoforms sequences) containing 125,800 protein sequence entries and the UniGene transcriptome database of *O. vulgaris* paralarvae [20] containing 77,838 protein sequence entries. The software used for the analysis was SEQUEST-HT (Protein Discoverer 2.4, Thermo Fisher Scientific). The following restrictions were used: tryptic cleavage with up to two missed cleavage sites and tolerances of 10 ppm for parent ions and 0.06 Da for MS/MS fragment ions. Carbamidomethylation of Cys (C*) was considered as a fixed modification. The permissible variable modifications were methionine oxidation (Mox) and acetylation of the N-terminus of the protein (N-Acyl). The results were subjected to statistical analysis with the Percolator algorithm to keep the false discovery rate (FDR) below 1%. For relative protein abundance determination for each sample, a label-free quantification (LFQ) method was used applying the Minora Feature Detector node and the ANOVA (individual proteins) method included in the Proteome Discoverer 2.4 software (Thermo Fisher Scientific). Peak areas of ion features from the same peptide for different charge forms were accumulated to one value. All the proteins obtained by the different analyses were used to create a reference dataset of mucus proteomes.

### 4.8. Euclidean Hierarchical Clustering

The function heatmap.2 of the statistical package R version (v) 4.1.1 (http://www.r-project.org, accessed on 20 March 2023) was used to achieve the Euclidean hierarchical clustering of the data. The Ggplots v.4.1.1 package, the Euclidean distance metric plus the complete linkage for the agglomeration method were performed as constraints.

### 4.9. Functional Gene Ontologies and Pathways Analysis

The final list of non-redundant protein Gene IDs were submitted to the PANTHER program version 17.0 (http://www.pantherdb.org, accessed on 4 June 2022), for the classification of proteins based on three main types of annotations: Molecular Function, Biological Process, and Protein Class. Statistical significance of the analysis representation was also provided.

The KEGG pathway analysis was performed by comparing the input data with the background of the *O. bimaculoides* genome by DAVID version 6.8 (https://david.ncifcrf.gov/home.jsp, accessed on 6 June 2022) [49]. The functional domains by InterPro Motifs were obtained comparing the input data with the background of the *O. vulgaris*, *O. bimaculoides, Lottia gigantea*, and *Crassostrea gigas* genomes using DAVID software (version 6.8).

### 4.10. Network Analysis

Network analysis was performed by running the protein dataset through the STRING (Search Tool for the Retrieval of Interacting Genes) software (v.11.0) (http://stringdb.org/, accessed on 8 June 2022) [50]. To minimize false positives as well as false negatives, all interactions tagged as “medium-confidence” (<0.7) in the STRING software have been eliminated from the analysis. Cluster networks were created using the MCL (Markov Cluster Algorithm) inflation algorithm, which is included in the STRING website and a value of 2 was selected for all the analyses.

### 4.11. Bioactive Peptides Prediction

Bioactive peptides encoded in the *O. vulgaris* skin mucus proteome was predicted by combining different in-silico protein hydrolysates using pepsin and trypsin enzymes. For this purpose, all proteolytic digestions were performed in-silico using the MS-Digest software, which is included on the ProteinProspector v.6.4.0 website (https://prospector.ucsf.edu/prospector/mshome.htm, accessed on 10 June 2022).

To analyze the results, all peptides were ranked using the PeptideRanker software (http://distilldeep.ucd.ie/PeptideRanker/, accessed on 10 June 2022) using the N-to-1 neural network probability to predict which peptides can be more bioactive [15]. Selected peptides with a score of >0.9 in PeptideRanker were compared with the BIOPEP-UWM database (http://www.uwm.edu.pl/biochemia/index.php/pl/biopep/, accessed on 19 June 2022) [51] and CAMP database (http://www.bicnirrh.res.in/antimicrobial/, accessed on 19 June 2022) [52] applying the DAC score (Discriminate Analysis Classifier score) to predict all potential bioactive peptides.

## 5. Conclusions

The results support the role of *O. vulgaris* skin mucus as a first line of interaction between the animal and its environment, indicating the importance of mucus in the regulation of the homeostasis of the animal and its importance as a barrier against physical, chemical, and biological aggressions from the immediate environment. *O. vulgaris* skin mucus showed a high protein richness, with a significant presence of proteins related to the immune response (histones, leucine-rich repeat immunoglobulin-like domain-containing nogo receptor-interacting protein, and factor H-like protein) and with a large number of potential bioactive peptides, which might play an important role in the antimicrobial response (mucins, hemocyanin, titin peptides, among others) and with potential uses in the fields of the biomedicine, pharmaceutical, and nutraceutical industries. Moreover, many of the uncharacterized proteins could have great potential to contain antimicrobial bioactive peptides, emphasizing the need for increased study in this area.

## Figures and Tables

**Table 1:** Protein concentration estimate with the BCA method for six specimens (O1–O6) of *O. vulgaris* skin mucus.

Sample	Absorbance	Concentration µg/µL
O1	0.67	2.43
O2	0.43	1.44
O3	0.18	0.42
O4	0.10	0.07
O5	0.14	0.23
O6	0.18	0.42

**Table 2:** KEGG pathway analysis of the *O. vulgaris* skin mucus proteome by DAVID (https://david.ncifcrf.gov/home.jsp, accessed on 6 June 2022).

KEGG Pathway	*p*-Value
Ribosome	3.90 × 10^−2^
Phagosome	8.30 × 10^−2^

**Table 3:** Functional InterPro motifs analysis of the *O. vulgaris* skin mucus proteome by DAVID (https://david.ncifcrf.gov/home.jsp, accessed on 6 June 2022).

InterPro Motifs	Count	%	*p*-Value
von Willebrand factor, type D domain	11	2.66	5.46 × 10^−9^
Lamin Tail Domain	7	1.69	1.83 × 10^−7^
Lipid transport protein, N-terminal	6	1.45	4.46 × 10^−7^
Lipid transport protein, beta-sheet shell	6	1.45	9.54 × 10^−7^
Glycoside hydrolase, superfamily	11	2.66	1.34 × 10^−6^
Metallopeptidase, catalytic domain	9	2.18	4.34 × 10^−5^
Uncharacterized domain, cysteine-rich	6	1.45	7.74 × 10^−5^
von Willebrand factor, type A	11	2.66	1.08 × 10^−4^
EF-hand domain	13	3.15	2.77 × 10^−4^
Vitellinogen, beta-sheet N-terminal	4	0.97	3.50 × 10^−4^
Vitellinogen, superhelical	4	0.97	4.51 × 10^−4^
FAS1 domain	4	0.97	4.51 × 10^−4^
Speract/scavenger receptor-related	5	1.21	5.00 × 10^−4^
EF-hand-like domain	14	3.39	6.06 × 10^−4^
Speract/scavenger receptor	5	1.21	6.38 × 10^−4^
Intermediate filament protein, conserved site	4	0.97	8.59 × 10^−4^
Chitin binding domain	9	2.18	1.09 × 10^−3^
Thrombospondin, type 1 repeat	6	1.45	1.28 × 10^−3^
Trypsin Inhibitor-like, cysteine rich domain	4	0.97	1.45 × 10^−3^
Peptidase M12A, astacin	4	0.97	1.69 × 10^−3^
EF-Hand 1, calcium-binding site	11	2.66	1.74 × 10^−3^
Concanavalin A-like lectin/glucanase, subgroup	9	2.18	1.80 × 10^−3^
MAM domain	4	0.97	3.65 × 10^−3^
Vitellinogen, open beta-sheet	3	0.73	3.8 × 10^−3^
Vitellinogen, open beta-sheet, subdomain 1	3	0.73	3.8 × 10^−3^
MD-2-related lipid-recognition (ML) domain	3	0.73	3.85 × 10^−3^
Low-density lipoprotein (LDL) receptor class A repeat	6	1.45	4.4 × 10^−3^
Carbonic anhydrase, alpha-class, conserved site	3	0.73	4.91 × 10^−3^
Actin-related protein	4	0.97	6.04 × 10^−3^
Actin, conserved site	3	0.73	6.09 × 10^−3^
Actinin-type, actin-binding, conserved site	4	0.97	6.60 × 10^−3^
Peptidase, metallopeptidase	4	0.97	7.20 × 10^−3^
Alpha carbonic anhydrase	3	0.73	7.38 × 10^−3^
Carbonic anhydrase, alpha-class	3	0.73	7.38 × 10^−3^
Glycoside hydrolase family 20, catalytic core	3	0.73	7.38 × 10^−3^
Beta-hexosaminidase subunit alpha/beta	3	0.73	7.38 × 10^−3^
Peptidase C14, caspase non-catalytic subunit p10	5	1.21	7.40 × 10^−3^
Peptidase C14, caspase precursor p45, core	5	1.21	7.82 × 10^−3^
Peptidase C14, ICE, catalytic subunit p20	5	1.21	8.24 × 10^−3^
Peptidase C14, caspase precursor p45	5	1.21	9.14 × 10^−3^
EGF domain, merozoite surface protein 1-like	3	0.73	1.19 × 10^−2^
Translation elongation factor EFTu/EF1A, domain 2	3	0.73	1.19 × 10^−2^
Low-density lipoprotein (LDL) receptor class A, conserved site	5	1.21	1.28 × 10^−2^
Cystine knot, C-terminal	3	0.73	1.37 × 10^−2^
Chitinase II	4	0.97	1.68 × 10^−2^
Villin/Gelsolin	3	0.73	1.74 × 10^−2^
Glycoside hydrolase, family 18, catalytic domain	4	0.97	2.00 × 10^−2^
Aldehyde dehydrogenase, C-terminal	3	0.73	2.38 × 10^−2^
Myosin tail	3	0.73	2.38 × 10^−2^
Gelsolin domain	3	0.73	2.60 × 10^−2^
Aldehyde dehydrogenase domain	3	0.73	2.60 × 10^−2^
Aldehyde/histidinol dehydrogenase	3	0.73	2.60 × 10^−2^
Aldehyde dehydrogenase, N-terminal	3	0.73	2.60 × 10^−2^
Uncharacterized domain, di-copper center	3	0.73	2.84 × 10^−2^
Tyrosinase	3	0.73	2.84 × 10^−2^
Spectrin repeat	4	0.97	3.72 × 10^−2^
Spectrin/alpha-actinin	4	0.97	3.87 × 10^−2^
Peptidase C14, ICE, catalytic subunit p20, active site	3	0.73	3.88 × 10^−2^
Epidermal growth factor-like domain	10	2.42	3.95 × 10^−2^
Epidermal growth factor-like domain	10	2.42	3.95 × 10^−2^
Elongation factor, GTP-binding domain	3	0.73	4.15 × 10^−2^
Cyclophilin-like peptidyl-prolyl cis-trans isomerase domain	3	0.73	4.44 × 10^−2^
Calponin homology domain	6	1.45	4.55 × 10^−2^
Glycoside hydrolase, chitinase active site	3	0.73	7.29 × 10^−2^
Translation elongation/initiation factor/Ribosomal, beta-barrel	3	0.73	7.29 × 10^−2^
EGF-like calcium-binding, conserved site	5	1.21	7.54 × 10^−2^
Peptidase S1, trypsin family, active site	4	0.97	7.64 × 10^−2^
Peptidase S1	4	0.97	9.46 × 10^−2^
Trypsin-like cysteine/serine peptidase domain	4	0.97	1.02 × 10^−1^
EGF-like calcium-binding	6	1.45	1.45 × 10^−1^
Insulin-like growth factor binding protein, N-terminal	5	1.21	1.58 × 10^−1^
EGF-type aspartate/asparagine hydroxylation site	5	1.21	1.72 × 10^−1^
WD40 repeat, conserved site	5	1.21	2.11 × 10^−1^
Leucine-rich repeat, typical subtype	6	1.45	2.11 × 10^−1^
Sushi/SCR/CCP	3	0.73	2.19 × 10^−1^
Myosin head, motor domain	3	0.73	2.55 × 10^−1^
Apple-like	3	0.73	3.39 × 10^−1^
IQ motif, EF-hand binding site	3	0.73	4.41 × 10^−1^
Leucine-rich repeat	6	1.45	4.64 × 10^−1^
Immunoglobulin-like domain	5	1.21	5.68 × 10^−1^
WD40 repeat	6	1.45	6.24 × 10^−1^
Fibronectin, type III	3	0.73	7.21 × 10^−1^
WD40/YVTN repeat-like-containing domain	6	1.45	7.23 × 10^−1^
Immunoglobulin I-set	3	0.73	7.90 × 10^−1^
Death domain	3	0.73	8.03 × 10^−1^
Death-like domain	4	0.97	8.12 × 10^−1^
Src homology-3 domain	4	0.97	8.60 × 10^−1^
Immunoglobulin subtype	3	0.73	8.67 × 10^−1^
Ankyrin repeat-containing domain	3	0.73	9.96 × 10^−1^
Ankyrin repeat	3	0.73	9.99 × 10^−1^

## Data Availability

Data available in a publicly accessible repository. The data presented in this study are openly available in [MassIVE, (https://massive.ucsd.edu/, accessed on 15 July 2022)], reference number [MSV000089888] and [ProteomeXchange database, (https://www.proteomexchange.org/, accessed on 15 July 2022)] reference number [PXD035356]. The original data presented in the study are also included in the article/Supplementary Material; further inquiries can be directed to the corresponding authors.

## Supplementary Materials

The supporting information can be downloaded at: https://www.mdpi.com/article/10.3390/ijms24087145/s1.
