# Supplementary material for: Identifying Natural Bioactive Peptides from the Common Octopus (Octopus vulgaris Cuvier, 1797) Skin Mucus By-Products Using Proteogenomic Analysis
Source: Int J Mol Sci. 2023 Apr 12;24(8):7145. doi: 10.3390/ijms24087145 (PMC10138644; doi:10.3390/ijms24087145)
Supplement: Supplementary file 1 [file ijms-24-07145-s001.zip › Figures S1 and S2.pdf]

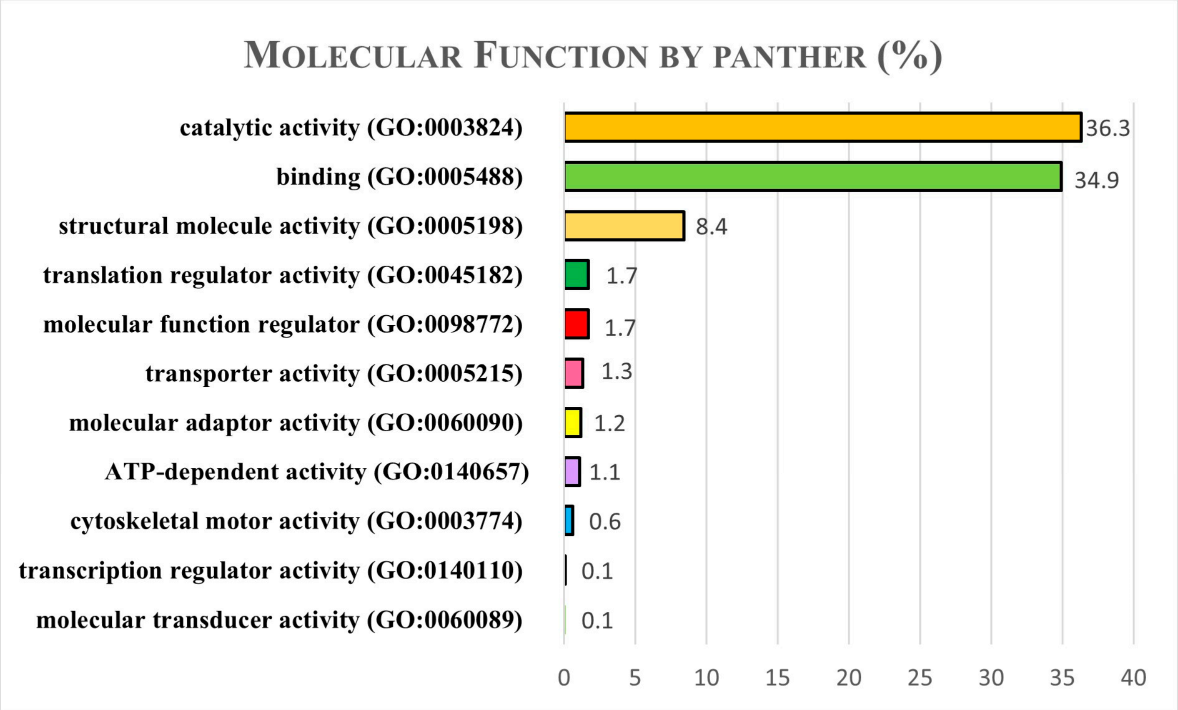

Figure S1: Molecular function of *O. vulgaris* skin mucus proteome identified by shotgun proteomics and categorized by PANTHER using the gene name as input for the software (http://pantherdb.org).

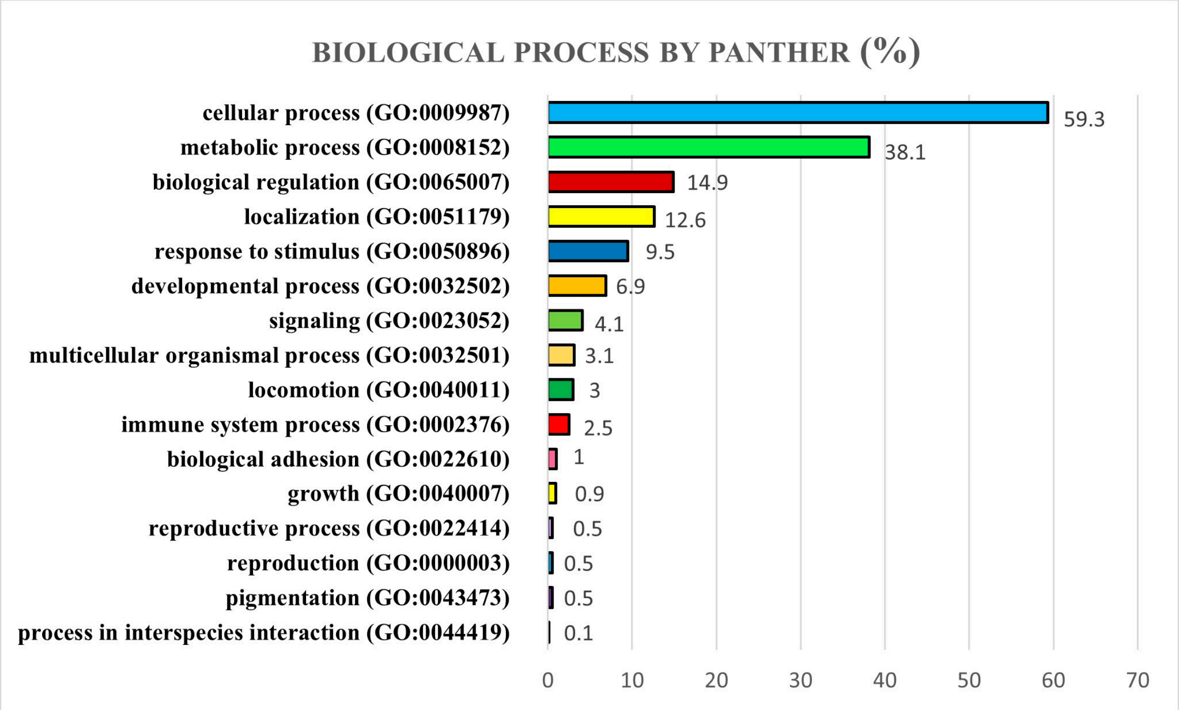

Figure S2: Biological process of *O. vulgaris* skin mucus proteome identified by shotgun proteomics and categorized by PANTHER using the gene name as input for the software (http://pantherdb.org).
